# Impact of zinc deficiency on mortality risk in patients with chronic obstructive pulmonary disease: a retrospective analysis

**DOI:** 10.3389/fnut.2025.1655272

**Published:** 2025-10-10

**Authors:** I-Wen Chen, Ting-Sian Yu, Yi-Chen Lai, Ping-Hsin Liu, Ying-Jen Chang, Jheng-Yan Wu, Kuo-Chuan Hung

**Affiliations:** ^1^Department of Anesthesiology, Chi Mei Medical Center, Liouying, Tainan City, Taiwan; ^2^Department of Anesthesiology, E-Da Hospital, I-Shou University, Kaohsiung City, Taiwan; ^3^Department of Anesthesiology, Chi Mei Medical Center, Tainan City, Taiwan; ^4^Department of Nutrition, Chi Mei Medical Center, Tainan City, Taiwan

**Keywords:** zinc deficiency, chronic obstructive pulmonary disease, mortality, prognosis, pneumonia

## Abstract

**Background:**

Chronic obstructive pulmonary disease (COPD) is a leading cause of morbidity and mortality worldwide. Although nutritional deficiencies are increasingly recognized as modifiable factors in COPD progression, the relationship between zinc status and clinical outcomes remains poorly understood. This study examined the association between zinc deficiency and clinical outcomes in patients with COPD.

**Methods:**

We conducted a retrospective cohort study using the TriNetX Research Network to analyze patients aged ≥18 years with established COPD, who underwent zinc testing between January 2010 and June 2023. Patients were categorized into zinc deficiency (serum zinc <70 μg/dL) and control groups (70–120 μg/dL). Using 1:1 propensity score matching, we created balanced cohorts controlling for demographics, comorbidities, and medications. The primary outcome was 6-month mortality, whereas secondary outcomes included COPD exacerbations, pneumonia, intensive care unit (ICU) admissions, hospital admissions, and hyperglycemic episodes. We also analyzed the impact of severe zinc deficiency (<50 μg/dL) and high zinc levels (>120 μg/dL) on prognosis.

**Results:**

After matching, 3,525 patients were included in each group. Zinc deficiency was associated with a significantly increased 6-month mortality risk (hazard ratio [HR]: 1.94, 95% confidence interval [CI]: 1.49–2.52, *p* < 0.001). The secondary outcomes demonstrated consistent patterns of increased morbidity, including higher risks of pneumonia (HR 1.24; *p* = 0.031), hyperglycemia (HR 1.28; *p* < 0.001), ICU admission (HR 1.61; *p* < 0.001), and hospital admission (HR 1.28; *p* < 0.001), with no significant difference in the risk of COPD exacerbations. Severe zinc deficiency exhibited dose-dependent effects, with markedly elevated risks across all outcomes. Interestingly, high zinc levels were also associated with increased mortality (HR, 1.74; *p* = 0.005), suggesting a U-shaped relationship between zinc status and mortality risk.

**Conclusion:**

Zinc deficiency represents a significant and independent risk factor for mortality and morbidity in patients with COPD, with evidence of dose-dependent effects and a U-shaped risk relationship. These findings suggest that assessing and optimizing zinc status may represent an important yet under-recognized component of comprehensive COPD management strategies.

## 1 Introduction

Chronic obstructive pulmonary disease (COPD) is one of the leading causes of morbidity and mortality worldwide, affecting over 300 million individuals globally and imposing substantial healthcare burdens through frequent hospitalizations, acute exacerbations, and progressive functional decline (1–4). Beyond the well-established risk factors of smoking and environmental exposure (5, 6), emerging evidence suggests that nutritional deficiencies may play a crucial yet underappreciated role in COPD progression and outcomes (7–9). Malnutrition affects approximately 17–30% (10–12) of COPD patients depending on disease severity, creating a complex interplay between respiratory function, systemic inflammation, and metabolic dysfunction that can accelerate disease progression and increase mortality risk (7, 10, 13). Among the various micronutrient deficiencies observed in COPD populations, zinc deficiency has garnered particular attention owing to its fundamental roles in immune function, antioxidant defense, and cellular repair mechanisms that are critically important for respiratory health (8, 14–16). Zinc may influence COPD outcomes through several key mechanisms. As a cofactor for antioxidant enzymes, such as superoxide dismutase, it helps reduce oxidative stress, a major driver of airway damage. Zinc also regulates immune responses and maintains epithelial barrier integrity, and its interaction with nutritional status and systemic inflammation further shapes COPD progression. Therefore, zinc deficiency can exacerbate inflammation and metabolic dysregulation, contributing to poorer outcomes in COPD.

Zinc serves as a cofactor for over 300 enzymes and plays essential roles in multiple biological processes directly relevant to COPD pathophysiology, including immune system regulation, wound healing, protein synthesis, and antioxidant enzyme function (8, 14–16). In the respiratory system, zinc deficiency can impair alveolar epithelial cell integrity, reduce surfactant production, and compromise the body’s ability to combat respiratory infections and oxidative stress (17–20). Previous studies have suggested associations between low zinc levels and increased susceptibility to respiratory tract infections, impaired lung function, and prolonged recovery from acute illness (21, 22); however, the relationship between zinc status and clinical outcomes in COPD patients remains incompletely understood. Furthermore, although zinc supplementation has become increasingly popular, particularly following the COVID-19 pandemic (23, 24), the optimal zinc levels for patients with COPD and the potential risks of excessive zinc intake have not been systematically investigated. This knowledge gap is particularly concerning given that both zinc deficiency and excess can have adverse health effects (25), suggesting a narrow therapeutic window that requires careful clinical consideration.

To address these critical knowledge gaps, this study aimed to examine the optimal zinc range for patients with COPD and provide evidence-based guidance for clinical practice. Given the growing use of zinc supplementation in respiratory conditions and the potential for both beneficial and harmful effects, understanding the precise relationship between zinc status and outcomes has immediate clinical relevance for improving patient care, reducing hospitalizations, and potentially decreasing mortality in this vulnerable population.

## 2 Methods

### 2.1 Data sources

This retrospective study utilized data from the TriNetX Research Network, a federated health research network that provides access to electronic health records from healthcare organizations worldwide. The platform provides access to real-world clinical data encompassing comprehensive patient information, including demographic characteristics, laboratory values, diagnoses coded using the International Classification of Diseases, Tenth Revision, Clinical Modification (ICD-10-CM), medical procedures, and prescribed medications, with standardized drug coding systems. The TriNetX database has been extensively used in numerous peer-reviewed publications, supporting its reliability as a source of real-world evidence (26–28). The study was approved by the Institutional Review Board of Chi Mei Medical Center (IRB number: 11310-E04), which waived the requirement for informed consent owing to the retrospective nature of the study and the use of de-identified data, which posed minimal risk to the participants.

### 2.2 Study population and eligibility criteria

The study population comprised patients aged ≥18 years who underwent zinc testing between January 1, 2010, and June 30, 2023. Participants were categorized into two distinct groups based on their serum zinc concentrations: the zinc deficiency group (ZD group) included patients with serum zinc levels below 70 μg/dL, while the control group consisted of patients with serum zinc levels between 70–120 μg/dL, representing the widely accepted normal physiological range (16, 25). Accordingly, values >120 μg/dL were classified as high zinc levels, reflecting concentrations above the upper normal limit. The date of zinc testing served as the index date for each patient, establishing a clear temporal reference point for subsequent outcome assessment and ensuring uniform follow-up periods across the study cohort.

All eligible patients must have had an established diagnosis of COPD documented prior to the index date, ensuring that the study population represented individuals with existing respiratory compromise, who might be particularly vulnerable to zinc deficiency-related complications. The diagnosis of COPD in this study was identified using the ICD-10-CM codes documented in the electronic health records of participating institutions. While the GOLD guidelines provide standardized clinical and spirometric criteria for COPD diagnosis, our retrospective design necessitated reliance on ICD-10 coding to ensure consistent identification of patients across the TriNetX network.

### 2.3 Exclusion criteria

To ensure a homogeneous study population and minimize confounding variables, patients with a pre-existing history of end-stage renal disease (ESRD) or chronic kidney disease stage 4 or 5 were excluded to avoid the confounding effects of advanced renal dysfunction on both zinc metabolism and clinical outcomes. Additionally, patients with a history of hemodialysis before the index date were excluded, as dialysis can significantly alter zinc homeostasis and overall prognosis. We also excluded patients with a documented history of malignant neoplasms of the bronchus and lung, heart or lung transplantation, cirrhosis of the liver, or respiratory tuberculosis prior to the index date, as these conditions could independently influence both the zinc status and respiratory outcomes. Furthermore, to eliminate the potential impact of acute illness on zinc levels, we excluded patients who experienced acute conditions within 1 month before the index date, including acute kidney injury, pneumonia, COPD with acute exacerbation, and sepsis.

### 2.4 Data collection and matching strategy

To minimize selection bias and ensure a balanced comparison between the groups, we applied a 1:1 propensity score-matching approach. This strategy incorporated not only demographic and clinical variables but also detailed laboratory and therapeutic data potentially affecting zinc status and clinical outcomes. Baseline characteristics were extracted from the 3 years prior to the index date and included age, sex, race, body mass index (BMI), serum albumin, hemoglobin A1c (HbA1c), and hemoglobin levels.

The Global Initiative for Chronic Obstructive Lung Disease (GOLD) stage was not available in the database, and FEV₁ values were infrequently recorded, making it difficult to incorporate direct COPD severity measures into matching. To minimize confounding by COPD severity and disease progression, we additionally matched patients for the presence of COPD-related complications, including the use of supplemental oxygen, history of COPD exacerbations, and presence of pulmonary hypertension. Furthermore, we controlled for the use of therapeutic agents that may affect COPD prognosis, specifically bronchodilators, glucagon-like peptide-1 receptor agonists (GLP-1 RAs), and sodium-glucose cotransporter-2 inhibitors (SGLT2is), which could potentially modify disease outcomes independent of the zinc status.

To further address potential treatment bias and ensure comparable therapeutic backgrounds, we matched for zinc supplementation use, as well as the utilization of angiotensin-converting enzyme (ACE) inhibitors and angiotensin II receptor blockers (ARBs), medications that could influence both renal function and overall cardiovascular health. Although C-reactive protein (CRP) levels are available in the TriNetX database, these measurements are not consistently obtained simultaneously with zinc testing. Although zinc concentrations can be transiently influenced by systemic inflammation, the lack of temporal alignment between CRP and zinc assessments could have introduced misclassification. Therefore, CRP was not incorporated as a covariate in our matching strategy.

### 2.5 Study outcomes

The primary outcome was the risk of overall mortality at the 6-month follow-up. Secondary outcomes included the 6-month risk of incident COPD exacerbation, pneumonia occurrence, intensive care unit (ICU) admission, episodes of high glucose levels (defined as glucose concentrations exceeding 180 mg/dL), and hospital admission. Glucose levels and pneumonia were included as secondary outcomes, as zinc deficiency may contribute to hyperglycemia and increase susceptibility to respiratory infections such as pneumonia, a common complication in COPD. To examine the long-term effects of zinc deficiency on clinical outcomes and disease progression, we analyzed all outcomes at the 12-month follow-up.

### 2.6 Association of severe zinc deficiency and outcomes

To assess potential dose-dependent effects, we conducted a separate analysis comparing patients with severe zinc deficiency (serum zinc ≤ 50 μg/dL) to those with normal zinc levels (70–120 μg/dL). This threshold represents a more profound degree of zinc depletion, which might be associated with more pronounced clinical consequences. All outcomes were evaluated at both the 6-month and 12-month follow-up periods to assess whether severe zinc deficiency demonstrated a dose-dependent relationship with adverse clinical outcomes.

### 2.7 Association of high zinc levels and clinical outcomes

As zinc status exhibits a U-shaped dose–response relationship in many biological systems, where both deficiency and excess can be harmful, we conducted a separate analysis examining patients with high zinc levels (≥120 μg/dL) compared to those with normal zinc levels (70–120 μg/dL) at both the 6-month and 12-month follow-up periods.

### 2.8 Subgroup analyses

To explore potential effect modifications and identify patient populations that might be at a particularly high risk for zinc deficiency-related complications, we performed prespecified subgroup analyses. These analyses were stratified by clinically relevant characteristics, including sex, presence or absence of hypertension, dyslipidemia, overweight/obesity status, asthma, and diabetes mellitus.

### 2.9 Statistical analysis

Baseline characteristics were summarized using appropriate descriptive statistics, with continuous variables presented as means with standard deviations and categorical variables expressed as frequencies with percentages. To achieve an optimal balance in baseline characteristics between the comparison groups, we implemented propensity score matching using a greedy nearest-neighbor algorithm with appropriate caliper settings. The quality of matching was assessed using standardized mean differences (SMD), with values less than 0.1 indicating adequate balance between groups, and visual inspection of propensity score distributions to ensure appropriate overlap.

Time-to-event outcomes were analyzed using the Kaplan–Meier method, with between-group differences assessed via the log-rank test to evaluate statistical significance. The association between zinc status and clinical outcomes was quantified using Cox proportional hazard regression models to calculate hazard ratios (HRs) with 95% confidence intervals (CIs) accounting for the time-to-event nature of the data. For subgroup analyses, the statistical significance of the differences between subgroups was evaluated by examining the confidence interval overlap. All statistical analyses were conducted using the TriNetX platform, with a two-sided *p*-value < 0.05 considered statistically significant for all comparisons.

## 3 Results

### 3.1 Patient selection and baseline characteristics

**Figure 1:**
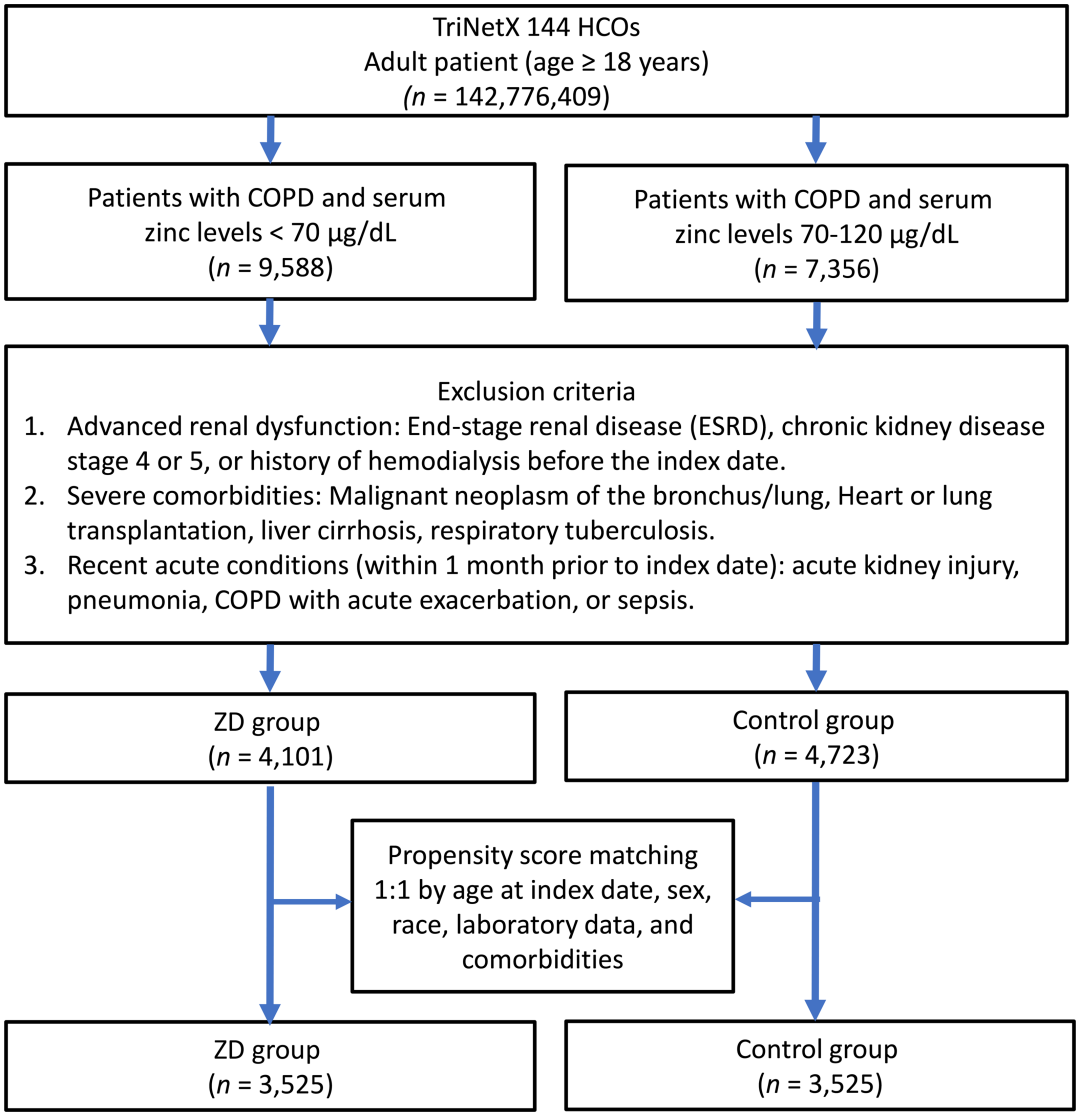
Patient selection flowchart from the TriNetX database. The diagram outlines the sequential exclusion criteria used to identify eligible patients with zinc deficiency (ZD) and those with normal zinc levels (control group). HCOs, Healthcare Organizations; COPD, chronic obstructive pulmonary disease.

**Figure 2:**
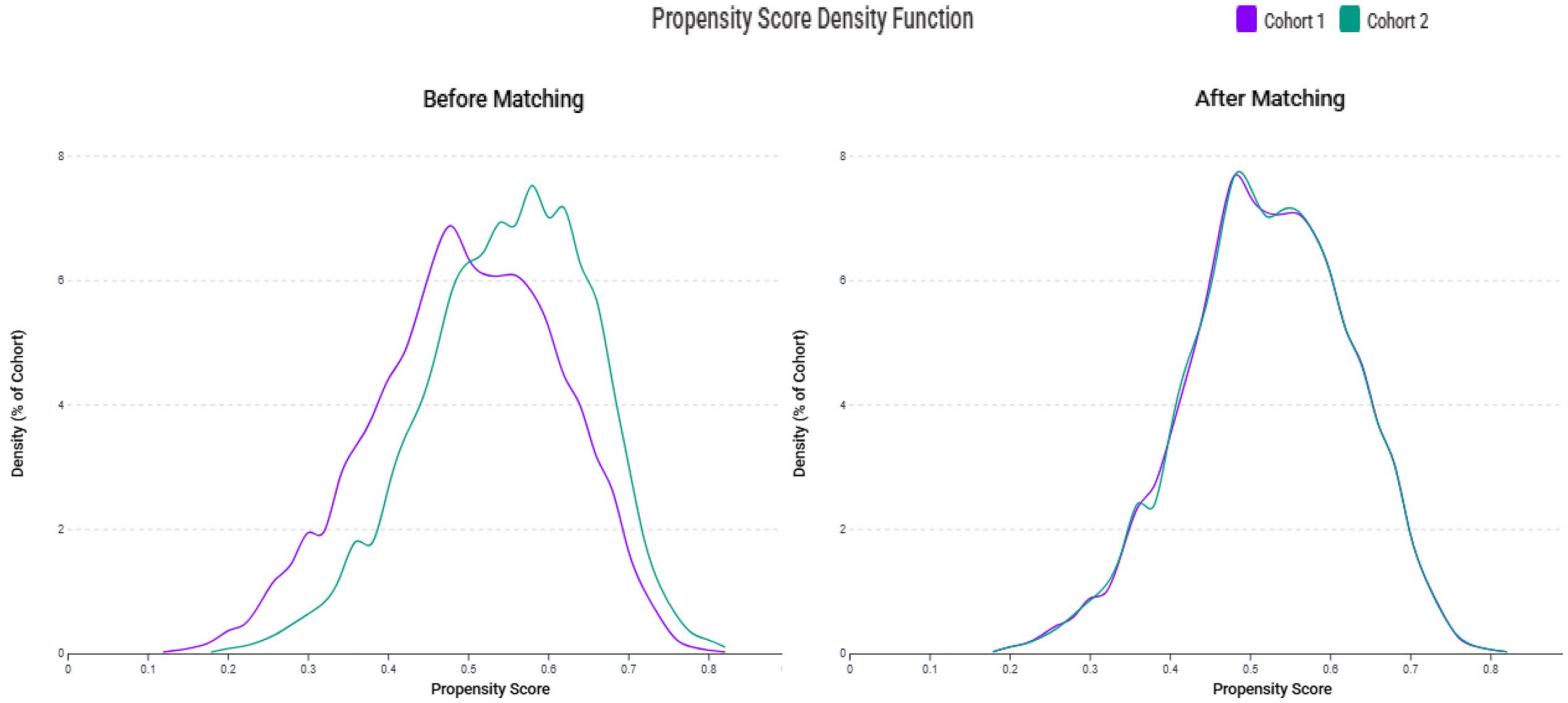
Propensity score density distributions before and after matching. The left panel shows distinct distribution patterns between the zinc deficiency group (Cohort 1) and the control group (Cohort 2) before matching. The right panel demonstrates improved overlap and covariate balance following 1:1 matching using a caliper of 0.1 standard deviations.

The systematic patient selection process is illustrated in Figure 1. Initially, the unmatched cohort comprised 4,101 patients in the zinc deficiency group and 4,723 patients in the control group. Through propensity score matching, we successfully created two well-balanced cohorts of 3,525 patients each, ensuring optimal comparability for subsequent analyses. The effectiveness of our matching strategy is visually demonstrated in Figure 2, where the propensity score density distributions show an improvement in the overlap between groups after matching.

Table 1 presents the baseline characteristics before and after propensity score matching. Prior to matching, several differences existed between groups, including body mass index (40.0 ± 10.6 kg/m^2^ in zinc deficiency versus 32.2 ± 10.2 kg/m^2^ in controls), dyslipidemia prevalence, overweight and obesity, anemia, heart failure, and malnutrition rates. After matching, these differences were effectively eliminated, with all standardized mean differences falling below 0.1, indicating excellent balance. The final matched cohorts were comparable across all key demographics, with a mean age of approximately 61 years, predominantly female composition (approximately 63%), and similar prevalence of major comorbidities, including diabetes mellitus (37%), hypertension (69%), and COPD-related complications.

**Table 1:** Baseline characteristics of patients before and after propensity score matching.

Variables	Before matching		After matching			
	ZD group (*n* = 4,101)	Control group (*n* = 4,723)	SMD†	ZD group (*n* = 3,525)	Control group (*n* = 3,525)	SMD†
Patient characteristics						
Age at index (years)	62.2 ± 14.1	60.8 ± 13.9	0.097	61.6 ± 14.2	61.5 ± 13.9	0.007
Female	2,533 (61.8%)	2,973 (62.9%)	0.024	2,226 (63.1%)	2,192 (62.2%)	0.020
BMI kg/m2	40.0 ± 10.6	32.2 ± 10.2	0.115	31.5 ± 10.4	31.8 ± 10.4	0.031
White	2,950 (71.9%)	3,278 (69.4%)	0.056	2,510 (71.2%)	2,479 (70.3%)	0.019
Comorbidities						
Essential (primary) hypertension	2,790 (68.0%)	3,245 (68.7%)	0.014	2,420 (68.7%)	2,419 (68.6%)	0.001
Dyslipidemia	2,248 (54.8%)	2,879 (61.0%)	0.125	2030 (57.6%)	2008 (57.0%)	0.013
Overweight and obesity	1754 (42.8%)	2,331 (49.4%)	0.132	1,631 (46.3%)	1,616 (45.8%)	0.009
Neoplasms	1,588 (38.7%)	1918 (40.6%)	0.039	1,392 (39.5%)	1,411 (40.0%)	0.011
Diabetes mellitus	1,480 (36.1%)	1758 (37.2%)	0.024	1,304 (37.0%)	1,318 (37.4%)	0.008
Asthma	1,366 (33.3%)	1770 (37.5%)	0.087	1,255 (35.6%)	1,265 (35.9%)	0.006
Ischemic heart diseases	1,404 (34.2%)	1,489 (31.5%)	0.058	1,169 (33.2%)	1,176 (33.4%)	0.004
Vitamin D deficiency	1,291 (31.5%)	1,631 (34.5%)	0.065	1,160 (32.9%)	1,174 (33.3%)	0.008
Other anemias	1,448 (35.3%)	1,371 (29.0%)	0.135	1,152 (32.7%)	1,141 (32.4%)	0.007
Nicotine dependence	1,366 (33.3%)	1,436 (30.4%)	0.062	1,112 (31.5%)	1,102 (31.3%)	0.006
Heart failure	1,008 (24.6%)	915 (19.4%)	0.126	791 (22.4%)	780 (22.1%)	0.007
Cerebrovascular diseases	686 (16.7%)	713 (15.1%)	0.045	571 (16.2%)	557 (15.8%)	0.011
Respiratory failure	740 (18.0%)	652 (13.8%)	0.116	568 (16.1%)	565 (16.0%)	0.002
Diseases of liver	682 (16.6%)	791 (16.7%)	0.003	565 (16.0%)	576 (16.3%)	0.008
COPD with exacerbation	668 (16.3%)	736 (15.6%)	0.019	562 (15.9%)	572 (16.2%)	0.008
Chronic kidney disease (CKD)	651 (15.9%)	626 (13.3%)	0.074	508 (14.4%)	510 (14.5%)	0.002
Malnutrition	766 (18.7%)	522 (11.1%)	0.216	476 (13.5%)	491 (13.9%)	0.012
Dependence on supplemental oxygen	390 (9.5%)	393 (8.3%)	0.042	324 (9.2%)	323 (9.2%)	0.001
Secondary pulmonary hypertension	412 (10.0%)	346 (7.3%)	0.097	308 (8.7%)	309 (8.8%)	0.001
Alcohol related disorders	456 (11.1%)	344 (7.3%)	0.133	296 (8.4%)	303 (8.6%)	0.007
COVID-19	267 (6.5%)	333 (7.1%)	0.021	240 (6.8%)	233 (6.6%)	0.008
Laboratory data						
Hemoglobin≥12 mg/dL	3,298 (80.4%)	4,056 (85.9%)	0.146	2,933 (83.2%)	2,922 (82.9%)	0.008
Hemoglobin A1c ≥ 7%	642 (15.7%)	782 (16.6%)	0.025	565 (16.0%)	571 (16.2%)	0.005
Albumin g/dL (≥3.5 g/dL)	3,258 (79.4%)	4,038 (85.5%)	0.160	2,927 (83.0%)	2,913 (82.6%)	0.011
FEV1 < 50%	53 (1.3%)	89 (1.9%)	0.047	50 (1.4%)	54 (1.5%)	0.009
Medications						
Antiasthma/bronchodilators	3,338 (81.4%)	3,905 (82.7%)	0.033	2,887 (81.9%)	2,882 (81.8%)	0.004
Antilipemic agents	1889 (46.1%)	2,255 (47.7%)	0.034	1,643 (46.6%)	1,645 (46.7%)	0.001
Antitussives/expectorants	1,143 (27.9%)	1,371 (29.0%)	0.026	1,005 (28.5%)	989 (28.1%)	0.010
Insulins and analogs	1,165 (28.4%)	1,240 (26.3%)	0.048	963 (27.3%)	980 (27.8%)	0.011
ACE inhibitors	1,047 (25.5%)	1,163 (24.6%)	0.021	905 (25.7%)	880 (25.0%)	0.016
Angiotensin II inhibitor	765 (18.7%)	932 (19.7%)	0.027	671 (19.0%)	669 (19.0%)	0.001
GLP-1 analogs	230 (5.6%)	345 (7.3%)	0.069	219 (6.2%)	217 (6.2%)	0.002
Zinc supplementation	238 (5.8%)	295 (6.2%)	0.019	200 (5.7%)	195 (5.5%)	0.006
SGLT2 inhibitors	107 (2.6%)	178 (3.8%)	0.066	102 (2.9%)	103 (2.9%)	0.002

ZD, zinc deficiency; BMI, body mass index; SMD, standardized mean differences; †SMD values <0.1 indicate adequate balance between groups; COPD, chronic obstructive pulmonary disease; GLP-1, Glucagon-like peptide-1; SGLT2, Sodium-glucose co-transporter 2; T2DM, Type 2 diabetes mellitus.

### 3.2 Association of zinc deficiency and 6-month outcomes

As shown in Table 2, zinc deficiency was associated with a nearly two-fold increase in 6-month mortality risk, with 163 deaths (4.6%) in the ZD group compared to 86 deaths (2.4%) in the control group (HR:1.94, 95% CI: 1.49–2.52, *p* < 0.001), underscoring the clinical relevance of zinc status in managing COPD. The secondary outcomes also demonstrated a consistent pattern of increased morbidity associated with zinc deficiency. Pneumonia risk was modestly but significantly elevated in the ZD group (HR 1.24, 95% CI: 1.02–1.50, *p* = 0.031), supporting the established role of zinc in immune function. Episodes of hyperglycemia (glucose >180 mg/dL) also occurred more frequently in zinc-deficient patients (HR 1.28, 95% CI: 1.13–1.43, *p* < 0.001), reflecting the crucial role of zinc in glucose metabolism. Regarding health source utilization, the risk of ICU admission (HR 1.61, 95% CI: 1.28–2.03, *p* < 0.001) and hospital admissions were also significantly increased (HR 1.28, 95% CI: 1.17–1.39, *p* < 0.001). Interestingly, there was no increased risk of COPD exacerbations in patients with zinc deficiency compared to the control group (HR 1.17, 95% CI: 0.95–1.45, *p* = 0.142).

**Table 2:** Association between zinc deficiency and 6-month outcomes.

Outcomes	ZD group (*n* = 3,525)	Control group (*n* = 3,525)	HR (95% CI)	*p*-value
	Events (%)	Events (%)		
Mortality	163 (4.6%)	86 (2.4%)	1.94 (1.49–2.52)	< 0.001
COPD with exacerbation	180 (5.1%)	157 (4.5%)	1.17 (0.95–1.45)	0.142
Pneumonia	230 (6.5%)	191 (5.4%)	1.24 (1.02–1.50)	0.031
Hyperglycemic episodes†	618 (17.5%)	505 (14.3%)	1.28 (1.13–1.43)	< 0.001
ICU admission	182 (5.2%)	116 (3.3%)	1.61(1.28–2.03)	< 0.001
Hospital admission	1,165 (33.1%)	974 (27.6%)	1.28 (1.17–1.39)	< 0.001

COPD, chronic obstructive pulmonary disease; †Glucose≥180 mg/dL; ICU, intensive care unit; ZD, zinc deficiency; HR, hazard ratio; CI, confidence interval.

### 3.3 Association of Zinc Deficiency and 12-month outcomes

Analysis at the 12-month follow-up (Table 3) demonstrated that the adverse effects of zinc deficiency persisted over extended follow-up periods, with a pattern remarkably similar to the 6-month findings. Mortality remained significantly elevated in the ZD group (HR 1.65, 95% CI: 1.34–2.03, *p* < 0.001), although with a somewhat attenuated effect size compared to the 6-month analysis. All other outcomes, including pneumonia (HR 1.19, *p* = 0.023), hyperglycemia (HR 1.23, *p* < 0.001), ICU admission (HR 1.61, *p* < 0.001), and hospital admission (HR 1.24, *p* < 0.001), showed statistically significant associations with zinc deficiency. The consistency of these findings across both time periods reinforces the sustained clinical impact of zinc status on COPD outcomes, suggesting that zinc deficiency is a persistent rather than a transient risk factor.

**Table 3:** Association between zinc deficiency and 12-month outcomes.

Outcomes	ZD group (*n* = 3,459)	Control group (*n* = 3,459)	HR (95% CI)	*p*-value
	Events (%)	Events (%)		
Mortality	234 (6.8%)	146 (4.2%)	1.65 (1.34–2.03)	< 0.001
COPD with exacerbation	279 (8.1%)	272 (7.9%)	1.05 (0.89–1.25)	0.540
Pneumonia	358 (10.4%)	310(9.0%)	1.19 (1.03–1.39)	0.023
Hyperglycemic episodes†	785 (22.7%)	666 (19.3%)	1.23 (1.11–1.37)	< 0.001
ICU admission	283 (8.2%)	182 (5.3%)	1.61 (1.34–1.94)	< 0.001
Hospital admission	1,501 (43.4%)	1,304 (37.7%)	1.24 (1.15–1.34)	< 0.001

COPD, chronic obstructive pulmonary disease; †glucose concentrations ≥180 mg/dL; ICU, intensive care unit; ZD, zinc deficiency; HR, hazard ratio; CI, confidence interval.

### 3.4 Dose-dependent effects: severe zinc deficiency analysis

The analysis of severe zinc deficiency revealed a dose–response relationship (Table 4), providing evidence for the biological plausibility of our findings. Patients with severe zinc deficiency demonstrated markedly elevated risks across all measured outcomes compared to those with normal zinc levels. The mortality risk was particularly pronounced at 6 months (HR: 1.96, 95% CI: 1.35–2.86, *p* < 0.001) and at 12 months (HR: 1.90, 95% CI: 1.41–2.55, *p* < 0.001).

**Table 4:** Association between severe zinc deficiency and outcomes.

Outcomes	6-m outcomes (*n* = 862 for each group)		12-m outcomes (*n* = 862 for each group)	
	HR (95% CI)	*p*-value*	HR (95% CI)	*p*-value*
Mortality	1.96 (1.35–2.86)	< 0.001	1.90 (1.41–2.55)	<0.001
COPD with exacerbation	2.42 (1.63–3.58)	< 0.001	1.87 (1.39–2.53)	<0.001
Pneumonia	1.72 (1.26–2.35)	< 0.001	1.88 (1.46–2.42)	<0.001
Hyperglycemic episodes†	1.90 (1.55–2.34)	< 0.001	1.80 (1.50–2.17)	<0.001
ICU admission	2.53 (1.77–3.62)	< 0.001	2.58 (1.93–3.45)	<0.001
Hospital admission	1.74 (1.49–2.03)	< 0.001	1.67 (1.46–1.92)	<0.001

COPD, chronic obstructive pulmonary disease; †glucose concentrations ≥180 mg/dL; ICU, intensive care unit; ZD, zinc deficiency; HR, hazard ratio; CI, confidence interval; *compared with the control group.

Most remarkably, severe zinc deficiency was associated with a more than two-fold increase in COPD exacerbation risk at 6 months (HR 2.42, 95% CI: 1.63–3.58, *p* < 0.001). This suggests that the relationship between zinc status and respiratory stability may have a critical threshold effect. ICU admission risks were dramatically elevated, with hazard ratios exceeding 2.5 at both time points. These findings collectively support a dose-dependent relationship between the severity of zinc deficiency and adverse clinical outcomes.

### 3.5 High zinc levels and clinical outcomes

The analysis of patients with elevated zinc levels revealed an important U-shaped relationship between zinc status and outcomes (Table 5). Contrary to the assumption that higher zinc levels might be protective, we observed significantly increased mortality risk in patients with high zinc levels compared to those with normal levels (HR: 1.74, 95% CI: 1.18–2.55, *p* = 0.005) at 6 months and at 12 months (HR: 1.79, 95% CI: 1.32–2.43, *p* < 0.001). ICU admission risk was also elevated in patients with high zinc status at the 6- and 12-m follow-up. In contrast, other outcomes, including COPD exacerbations, pneumonia, hyperglycemia, and hospital admissions, showed no significant associations with high zinc levels.

**Table 5:** Association between high zinc levels and outcomes.

Outcomes	6-m outcomes (*n* = 1,402 for each group)		12-m outcomes (*n* = 1,402 for each group)	
	HR (95% CI)	*p*-value*	HR (95% CI)	*p*-value*
Mortality	1.74 (1.18–2.55)	0.005	1.79 (1.32–2.43)	<0.001
COPD with exacerbation	1.04 (0.74–1.47)	0.820	1.15 (0.88–1.52)	0.313
Pneumonia	0.97 (0.72–1.30)	0.831	1.00 (0.79–1.28)	0.976
Hyperglycemic episodes†	1.11 (0.91–1.35)	0.301	1.17 (0.99–1.39)	0.074
ICU admission	1.67 (1.16–2.41)	0.005	1.44 (1.08–1.92)	0.013
Hospital admission	0.99 (0.86–1.15)	0.919	0.96 (0.85–1.09)	0.513

COPD, chronic obstructive pulmonary disease; †glucose concentrations ≥180 mg/dL; ICU, intensive care unit; ZD, zinc deficiency; HR, hazard ratio; CI, confidence interval; *compared with the control group.

### 3.6 Subgroup analysis

The subgroup analysis (Table 6) examined whether the impact of zinc deficiency on 6-month mortality varied across different patient populations. Although numerical differences in risk were observed between the subgroups, none of the interaction *p*-values reached statistical significance (all *p* > 0.05). These findings indicate that the adverse impact of zinc deficiency on mortality risk is consistent across sex, comorbidity status (hypertension, diabetes, dyslipidemia, obesity), and asthma presence, suggesting that the clinical importance of zinc status applies broadly to patients with COPD, regardless of their baseline characteristics.

**Table 6:** Subgroup analyses of association between zinc deficiency and risk of mortality at 6-m follow-up.

Subgroup analysis	Number of each group	HR (% CI)	*p*-value	P for interaction
Sex				
Male	1,053	2.01 (1.36–2.98)	<0.001	Reference
Female	2,252	1.92 (1.28–2.87)	0.001	0.877
Asthma				
Yes	1,308	1.64 (0.88–3.06)	0.116	Reference
No	2,164	1.93 (1.42–2.61)	<0.001	0.647
Obesity				
Yes	1,678	1.55 (0.92–2.58)	0.095	Reference
No	1,790	1.83 (1.35–2.48)	<0.001	0.585
Hypertension				
Yes	2,464	2.10 (1.53–2.89)	<0.001	Reference
No	1,031	1.23 (0.78–1.94)	0.368	0.056
Diabetes mellitus				
Yes	1,348	1.34 (0.86–2.09)	0.195	Reference
No	2,146	1.86 (1.35–2.57)	0.001	0.239
Dyslipidemia				
Yes	2,061	2.09 (1.45–3.01)	<0.001	Reference
No	1,408	1.58 (1.08–2.31)	0.017	0.314

HR, hazard ratio; CI, confidence interval.

### 3.7 Risk factors for 6-month mortality

Multivariable analysis (Table 7) confirmed zinc deficiency as an independent predictor of 6-month mortality after adjusting for multiple potential confounders. Zinc deficiency emerged as one of the strongest modifiable risk factors (HR: 2.03, 95% CI: 1.59–2.58, *p* < 0.001). Other significant independent predictors included male sex (HR 1.56, *p* < 0.001), advanced age (HR 1.05 per year, *p* < 0.001), heart failure (HR 2.54, *p* < 0.001), nicotine dependence (HR 1.44, *p* = 0.003), and malnutrition (HR 2.24, *p* < 0.001). Notably, overweight and obesity showed a protective effect (HR 0.54, 95% CI: 0.40–0.72, *p* < 0.001). The persistent association between zinc deficiency and adverse outcomes, even after adjusting for malnutrition, suggests that the effects of zinc extend beyond general nutritional status, underscoring its distinct biological role in COPD.

**Table 7:** Risk factors for mortality at 6-month follow up.

Variable	HR (95% CI)	*p* value†
Zinc deficiency vs. control	2.03 (1.59, 2.58)	<0.001
Male	1.56 (1.25, 1.95)	<0.001
Age at Index	1.05 (1.04, 1.06)	<0.001
Essential (primary) hypertension	0.90 (0.70, 1.16)	0.411
Overweight and obesity	0.54 (0.40, 0.72)	<0.001
Diabetes mellitus	0.87 (0.67, 1.13)	0.298
Heart failure	2.54 (1.98, 3.25)	<0.001
Ischemic heart diseases	0.86 (0.67, 1.11)	0.249
Chronic kidney disease (CKD)	0.86 (0.64, 1.15)	0.310
Nicotine dependence	1.44 (1.13, 1.83)	0.003
Malnutrition	2.24 (1.75, 2.87)	<0.001
Diseases of liver	1.11 (0.83, 1.50)	0.487
Other anemias	1.20 (0.95, 1.52)	0.121

HR, hazard ratio; CI, confidence interval; †multivariate logistic regression analysis.

## 4 Discussion

Our propensity-matched cohort study of over 7,000 patients with COPD demonstrated that zinc deficiency was independently associated with a substantially increased mortality risk, with effects persisting throughout both the short- and long-term follow-up periods. Beyond mortality, zinc deficiency has been consistently linked to increased morbidity across multiple clinically relevant outcomes, including respiratory infections, metabolic dysregulation, and healthcare resource utilization. Importantly, we identified a dose-dependent relationship, with severe zinc deficiency demonstrating even more pronounced adverse effects, while simultaneously revealing a U-shaped relationship, where excessively high zinc levels also conferred an increased mortality risk.

The nearly two-fold increase in mortality risk linked to zinc deficiency in our COPD population represents one of the most clinically significant findings in recent nutritional research on respiratory diseases. Our findings align with emerging evidence from other respiratory conditions and the general medical population. Previous studies on patients with COVID-19 have indicated an association between low zinc status and increased mortality (29, 30), whereas research on patients with heart failure has similarly demonstrated poor outcomes among zinc-deficient individuals (31). However, our study extends this knowledge by specifically focusing on patients with COPD, a population with unique metabolic demands and inflammatory burdens that may render them particularly vulnerable to zinc deficiency-related complications.

The dose-dependent relationship observed strengthens the biological plausibility of our findings. The fact that severe zinc deficiency demonstrated even stronger mortality associations suggests a threshold effect, where progressively lower zinc levels confer an incrementally higher risk. This gradient relationship supports a causal interpretation rather than merely reflecting confounding by general nutritional status or disease severity. The persistence of significant associations even after multivariate adjustment for malnutrition further reinforces the independent biological importance of zinc. Perhaps most intriguingly, our identification of a U-shaped relationship, where both deficiency and excess zinc levels increased mortality risk, reflects the known biological principle that zinc homeostasis requires precise regulation. This finding has immediate clinical implications, suggesting that indiscriminate zinc supplementation without monitoring may be potentially harmful, emphasizing the need for targeted, evidence-based approaches to zinc optimization in patients with COPD.

One of our most striking findings was the dramatic association between severe zinc deficiency and COPD exacerbations, with a more than two-fold increased risk observed in patients with the lowest zinc levels. This relationship is particularly noteworthy because mild-to-moderate zinc deficiency did not demonstrate significant associations with exacerbations, suggesting a critical threshold effect, in which only severe depletion compromises respiratory stability. This threshold phenomenon makes biological sense when considering the multifaceted roles of zinc in respiratory health. Zinc is essential for maintaining epithelial barrier integrity, supporting immune cell function, and regulating inflammatory responses (32). Under severely deficient conditions, these protective mechanisms may become sufficiently compromised to precipitate acute respiratory deterioration. The respiratory epithelium, which is already damaged in patients with COPD, may be particularly vulnerable to further compromise when zinc-dependent repair mechanisms are severely impaired. While routine zinc monitoring may not be cost-effective for all COPD patients, our results suggest that identifying and treating severe zinc deficiency could potentially reduce exacerbation rates, thereby improving the quality of life and reducing healthcare costs (33). Given that COPD exacerbations are major drivers of disease progression, disability, and healthcare expenditure, even modest reductions in exacerbation frequency could yield significant clinical and economic benefits.

Our findings regarding the increased risk of pneumonia and hyperglycemic episodes in zinc-deficient patients provide important mechanistic insights into how zinc deficiency contributes to poor outcomes. The increased risk of pneumonia aligns perfectly with the well-established immunomodulatory functions of zinc, particularly its role in T-cell development, macrophage function, and antimicrobial peptide production (34, 35). In patients with COPD who already face an increased risk of pneumonia due to impaired mucociliary clearance and chronic inflammation, zinc deficiency may represent an additional vulnerability that pushes their immune systems beyond compensatory capacity. The magnitude of the increased pneumonia risk, while modest, becomes clinically significant when considered at the population level, particularly given the role of pneumonia as a leading cause of COPD-related hospitalizations and mortality (36). However, we acknowledge that the elevated pneumonia risk observed in zinc-deficient patients may be attributable not only to the biological effects of zinc deficiency but also to the impact of COPD pharmacotherapy, particularly long-term inhaled corticosteroids use. Further studies are required to clarify this relationship. The association with hyperglycemic episodes illuminates another important pathway through which zinc deficiency may influence COPD outcomes. The crucial role of zinc in insulin synthesis, storage, and secretion indicates that zinc deficiency can directly impair glucose homeostasis (37). In patients with COPD who often receive corticosteroids and experience stress-induced hyperglycemia during acute illness, zinc deficiency may exacerbate glycemic dysregulation. Poor glucose control, in turn, can impair immune function, delay wound healing, and increase the risk of infection, creating a vicious cycle that compounds respiratory health problems.

The consistent mortality risk associated with Zn deficiency observed across the various patient subgroups enhances the generalizability of our findings. The absence of a significant effect modification by sex, comorbidity status, or other patient characteristics indicates that zinc deficiency is a universal risk factor that affects patients with COPD, regardless of their baseline clinical profile. This universal impact has important implications for clinical practice, suggesting that zinc assessment and optimization should be considered for all patients with COPD rather than targeting only specific high-risk subgroups. The multivariable analysis further reinforced the independent prognostic importance of zinc deficiency, demonstrating that its effects persist even after accounting for established mortality predictors, including age, sex, heart failure, and malnutrition. Interestingly, the protective effect of overweight and obesity observed in our analysis reflects the well-described “obesity paradox” in COPD (38, 39), where moderate excess weight appears beneficial, possibly due to metabolic reserves during acute illness or different inflammatory profiles. The persistence of adverse effects of zinc deficiency, even after accounting for this protective factor, highlights its distinct biological significance beyond general nutritional status.

Geographic variations in zinc deficiency may create different risk profiles for patients with COPD worldwide. While the prevalence is relatively low in high-income regions, it rises to 30% in South Asia and 24% in sub-Saharan Africa, with some countries reporting rates exceeding 70% (40, 41). These differences are largely driven by soil zinc content, dietary patterns, and food system characteristics, particularly high phytate-to-zinc ratios in cereal-based diets (40–42). For COPD patients, such regional disparities mean that the elevated mortality risk linked to zinc deficiency may have a far greater clinical impact in high-deficiency areas, underscoring the need for region-specific screening and supplementation strategies.

Several important limitations of our study must be acknowledged when interpreting our findings. First, the retrospective observational design, while enabling large-scale population analysis, cannot establish definitive causality between zinc deficiency and adverse outcomes. Second, we acknowledge that serum zinc was measured only once in this study, which may be influenced by acute illness, inflammatory states, or recent dietary intake, potentially leading to misclassification of zinc status. This limitation should be considered when interpreting our findings, although the large sample size and consistent results across outcomes support the robustness of our conclusions. Another limitation is that serum zinc levels were derived from routine clinical testing within the TriNetX network, where differences in assay methods, laboratory standards, and inter-laboratory variability may have introduced measurement inconsistencies. Third, we lacked information on zinc bioavailability, dietary zinc intake patterns, and the presence of factors that might impair zinc absorption, such as phytate consumption or gastrointestinal disorders. Fourth, because our data were derived from a healthcare network of institutions with electronic health records, the findings may not be fully generalizable to all patients with COPD in the general population. Finally, the exclusion of patients with certain comorbidities, while necessary to reduce confounding, may limit the applicability to the broader COPD population, which often includes individuals with multiple chronic conditions. Another limitation of our study is the absence of subgroup analyses stratified by COPD classification (Groups A, B, and E) and inhaler medication use. The lack of such analyses may limit insights into how zinc status interacts with disease severity or specific therapeutic regimens to shape outcomes.

## 5 Conclusion

Our results provide evidence that zinc deficiency represents a significant, modifiable risk factor for adverse outcomes in patients with COPD, with effects spanning mortality, respiratory stability, infectious complications, and metabolic control. The dose-dependent relationship observed, coupled with the identification of a U-shaped risk curve, suggests that optimal zinc status requires careful attention to both deficiency prevention and excess avoidance. These findings support the consideration of zinc assessment and targeted optimization as potential components of comprehensive COPD management strategies. Nevertheless, the optimal timing of zinc level testing remains uncertain. Given the dynamic influences of acute illness, inflammation, and nutritional fluctuations, prospective studies are needed to determine the most appropriate intervals for zinc monitoring in clinical practice. Until such evidence emerges, clinicians should maintain heightened awareness of zinc deficiency as a potentially important but under-recognized contributor to poor outcomes in COPD patients, particularly those with severe disease or frequent exacerbations.

## Data Availability

The raw data supporting the conclusions of this article will be made available by the authors, without undue reservation.
